# Functional and structural phenotyping of cardiomyocytes in the 3D organization of embryoid bodies exposed to arsenic trioxide

**DOI:** 10.1038/s41598-021-02590-8

**Published:** 2021-11-30

**Authors:** Paola Rebuzzini, Cinzia Civello, Lorenzo Fassina, Maurizio Zuccotti, Silvia Garagna

**Affiliations:** 1 Laboratory of Developmental Biology, Department of Biology and Biotechnology “Lazzaro Spallanzani”, University of Pavia, Via Ferrata 9, 27100 Pavia, Italy; 2 Department of Electrical, Computer and Biomedical Engineering (DIII), University of Pavia, Via Ferrata 5, Pavia, Italy; 3 Centre for Health Technologies (CHT), University of Pavia, Via Ferrata 5, Pavia, Italy

**Keywords:** Cell biology, Stem-cell differentiation, Environmental impact

## Abstract

Chronic exposure to environmental pollutants threatens human health. Arsenic, a world-wide diffused toxicant, is associated to cardiac pathology in the adult and to congenital heart defects in the foetus. Poorly known are its effects on perinatal cardiomyocytes. Here, bioinformatic image-analysis tools were coupled with cellular and molecular analyses to obtain functional and structural quantitative metrics of the impairment induced by 0.1, 0.5 or 1.0 µM arsenic trioxide exposure on the perinatal-like cardiomyocyte component of mouse embryoid bodies, within their 3D complex cell organization. With this approach, we quantified alterations to the (a) beating activity; (b) sarcomere organization (texture, edge, repetitiveness, height and width of the Z bands); (c) cardiomyocyte size and shape; (d) volume occupied by cardiomyocytes within the EBs. Sarcomere organization and cell morphology impairment are paralleled by differential expression of sarcomeric α-actin and Tropomyosin proteins and of *acta2*, *myh6* and *myh7* genes. Also, significant increase of *Cx40*, *Cx43* and *Cx45* connexin genes and of Cx43 protein expression profiles is paralleled by large Cx43 immunofluorescence signals. These results provide new insights into the role of arsenic in impairing cytoskeletal components of perinatal-like cardiomyocytes which, in turn, affect cell size, shape and beating capacity.

## Introduction

Chronic exposure to environmental pollutants can affect human health. One of the most diffused toxicants is arsenic, a metalloid element with ubiquitous distribution in the earth's crust, groundwater and biosphere^1^. The World Health Organization (WHO) estimates that over 200 million people worldwide are chronically exposed to arsenic through drinking water at concentrations above the WHO safety standard of 10 μg/L^2^. Contamination of drinking water is considered one of the top global health concerns, given the extent of population’s potential exposure and its association with several diseases, including diabetes, reproductive impairment, cancers and cardiac diseases^3,4^. In a recent study, arsenic exposure was related to an increase in left ventricular (LV) wall thickness and LV hypertrophy in young American Indians with low burden of cardiovascular risk factors^5^. Moreover, in a study aimed at investigating alterations of proteome profile in rat heart after long-term arsenic trioxide (ATO) exposure, 33 proteins, specifically involved in heart development, heart morphology, cardiac contraction and dilatation, were found significantly altered^6^.

Epidemiological studies of people exposed to arsenic via drinking water reported similar exposure levels in the foetus as in the mother, due to its capability to pass through the placental barrier^7^. In vivo arsenic exposure during cardiac development induces embryonic congenital heart defects and malformations in zebrafish^8^, chicken^9^ and rats^10,11^. Similarly, arsenic impairs mouse embryonic stem cells (mESCs) cardiomyocyte differentiation *in vitro*^12–14^, by reducing cardiomyocyte beating capacity^12,14^, proliferation^13^ and the expression of sarcomeric and connexin proteins^14^.

After birth, little arsenic is excreted in the breast milk^15^; instead, formula-prepared milk using drinking water may cause considerable post-natal exposure^16^. The effects of arsenic during the perinatal period are scarcely investigated and much remains to be learned about the mechanisms through which this pollutant induces functional and/or structural impairment of neonatal cardiomyocytes.

Perinatal-like cardiomyocytes (hereafter named cardiomyocytes) can be obtained by 15 days of in vitro differentiation of mESCs within the three-dimensional (3D) multi-cellular organization of the embryoid bodies (EBs)^17,18^. In the EBs, stochastic events lead to the induction of developmental cues that drive embryogenesis, such as the differentiation into derivatives of the three germ layers^19^, including spontaneously contracting cardiomyocytes. The 3D structure of the EBs represents a highly regulated environment in which gene expression, myofibrillar architecture, cellular ultrastructure and electrophysiological functions are controlled similarly to what occurs during cardiac development in vivo^20,21^. At the end of differentiation, the electrophysiological characteristics, sarcomere protein expression and ion channel expression of mESC-derived cardiomyocytes are reminiscent of embryonic and postnatal murine cardiomyocytes^17,22–24^.

In this study, we used this mESC differentiation platform to derive perinatal-like cardiomyocytes which were exposed to 0.1, 0.5 or 1.0 µM ATO, concentrations in the range of the inorganic arsenic present in contaminated water^25,26^, for 72 h. After treatments, EBs were video-recorded and, using a bioinformatic tool that we recently developed^14,24,27–29^, their kinematic parameters were computed to gain information on the cardiomyocyte contractile properties. Then, inside the 3D EBs organization, by combining sarcomere protein markers and confocal imaging with the SarcOmere Texture Analysis (SOTA) algorithm^30^, we quantified the sarcomere organization, the cardiomyocyte size and shape and the volume of the whole cardiomyocyte component. On the same EBs, Cx43 immunofluorescence was performed to localize gap junctions. In addition to this structural phenotyping analysis, the impact of ATO on cardiomyocytes was evaluated by the quantification of sarcomere- and gap-junction-specific genes and proteins.

## Results

### Kinematics and dynamics properties of cardiomyocytes

Within the 3D structure of the differentiating EBs, complex morphogenesis is recapitulated, leading to the acquisition of tissue-like structures, which, in our model, are characterized by spontaneous contractile activity, indicative of cardiomyocyte differentiation. Here, this activity was determined by image processing analysis of videos recording the contraction movement of beating EBs (Videos 1S, 2S, 3S and 4S)^14,24,27–29^. Specifically, the kinematic and dynamic cardiomyocyte features determined in a previous work of ours^27^, i.e. the chronotropy (beat frequency, [Hz]), dynamic inotropy (contraction force, [pixel/s^2^]), kinematic inotropy (contractility or maximum contraction velocity, [pixel/s]), and ergotropy (consumption of ATP for kinetic energy, [pixel^2^/s^2^]), were evaluated for comparison between untreated (CTR) and ATO-exposed samples.

Baseline CTR values of chronotropy and of kinematic inotropy were higher and lower, respectively, when compared to those obtained in a former work of ours^14^ where the differentiation process was halted at day 15. This was not completely surprising since in day 18 EBs, while the beating capacity of cardiac cells ameliorate, contractility might be affected by their dimension, which is much bigger than that of day 15 EBs.

**Figure 1:**
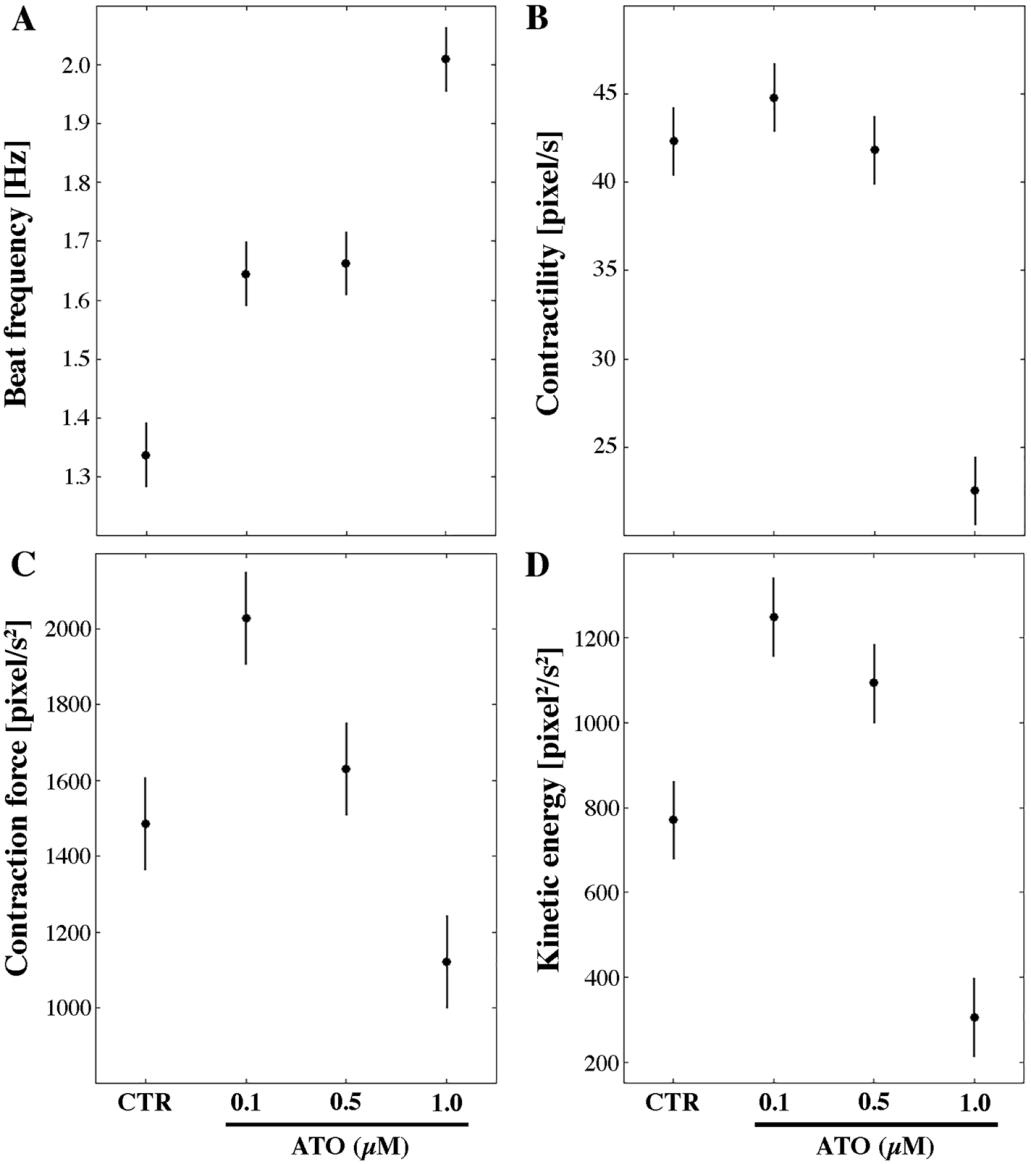
Contractile properties of beating areas within the EBs of control (CTR) and 0.1, 0.5 or 1.0 μM ATO exposed samples for 72 h. In A, beat frequency [Hz]; in B, contractility [pixel/s]; in C, contraction force [pixel/s^2^]; in D, kinetic energy [pixel^2^/s^2^]. The vertical bars represent 95% confidence intervals for the difference between averages according to the "Least Significant Difference" statistical test.

The presence of ATO, for 72 h at the end of the differentiation period, determined increase of the beat frequency (1.2-, 1.2-, 1.5-fold at 0.1, 0.5 and 1.0 µM, respectively; *p* < 0.05; Fig. 1A) and of the kinetic energy (1.6-, 1.4-fold at 0.1 and 0.5 µM, respectively; *p* < 0.05; Fig. 1D) at all concentrations with the exception, for the highest dose of a decrease in the kinetic energy (0.4-fold change, *p* < 0.05). Both cardiomyocytes contractility and contraction force diminished at the highest dose of 1.0 µM (0.5- and 0.7-fold, respectively; *p* < 0.05), while the latter parameter increased at the 0.1 µM dose (1.3-fold; *p* < 0.05) (Fig. 1B, C). No significant variation in contractility (*p* > 0.05) was observed after 0.1 or 0.5 µM ATO exposure or in the contraction force at 0.5 µM (*p* > 0.05).

Then, we verified whether altered kinematic and dynamic parameters are paralleled by changes in the mitochondrial activity. Evaluation of the ATP content showed no significant (*p* > 0.05) differences when comparing CTR to ATO-exposed cells at all concentrations tested (Fig. 1S), suggesting that the increased kinetic load does not affect ATP content.

Then, to understand whether these altered kinematics and dynamics properties may be paralleled by impairment of the sarcomere organization and of the gap junctions, quantitative metrics of sarcomeric cardiac α-actinin and immunolocalization of Cx43 protein were performed on confocal images obtained from EBs within their 3D context, in parallel with quantitation of sarcomeric and gap junction transcripts and proteins.

### Sarcomere texture analysis (SOTA)

**Figure 2:**
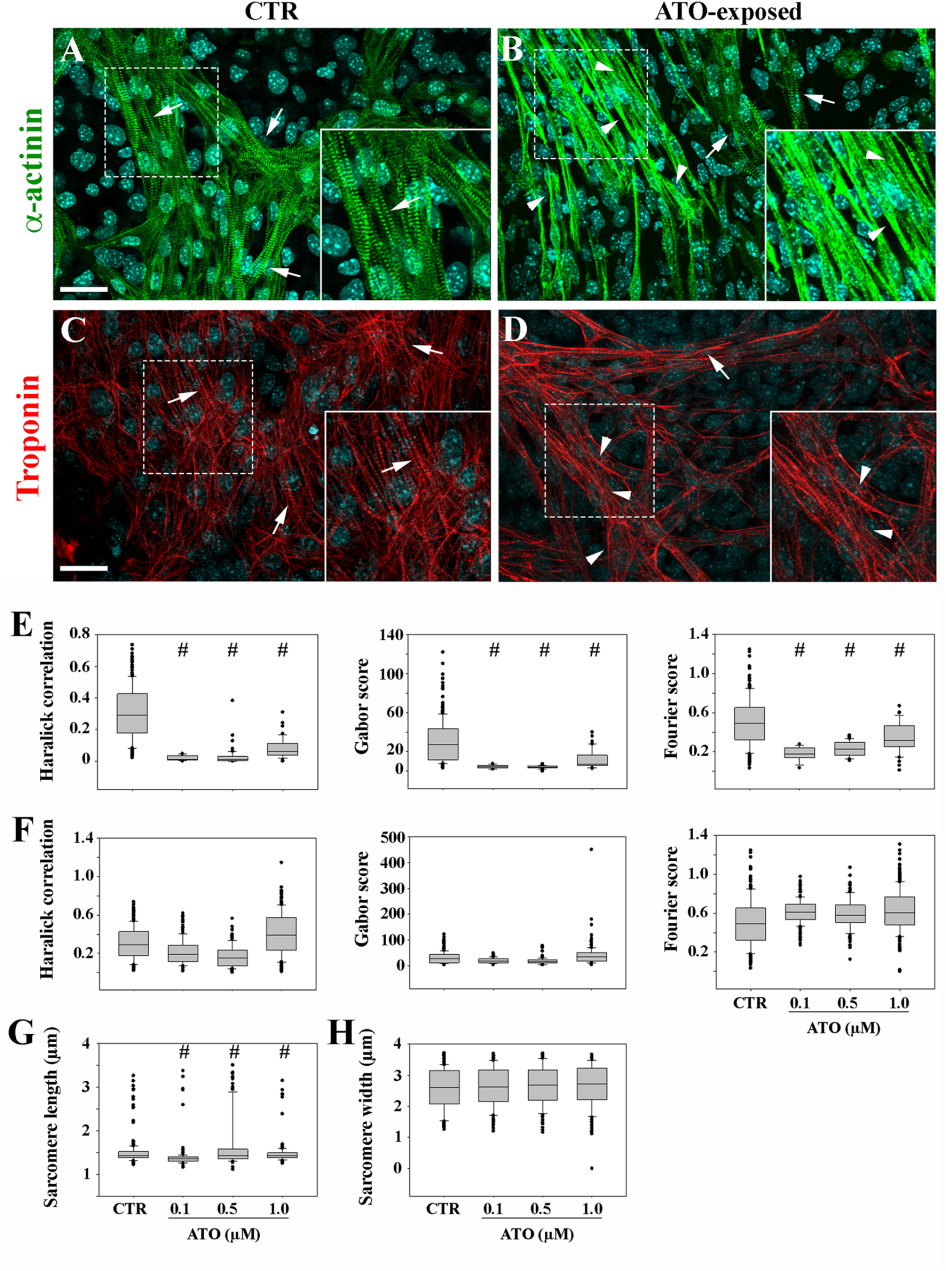
Whole mount immunofluorescence localization of cardiac α-actinin (**A** and **B**) and of Troponin T (**C** and **D**) in control (CTR) and in 0.1 µM ATO-exposed cardiomyocytes. White arrows point to correct sarcomere striated pattern (**A**, **C**, magnification in the insets); white arrowheads indicate disorganized sarcomeres (**B**, **D**) magnification in the insets). Bar: 20 μm. (**E**–**H**) Quantitative metrics of cardiomyocyte sarcomere structure. (**E**), the three scores refer to the comparison between organized sarcomeres of CTR samples and disorganized altered sarcomeres of ATO-exposed samples; (**F**) the comparison is made between organized sarcomeres present in both CTR and exposed samples. Length (**G**) and width (**H**) of organized sarcomeres in the comparison between CTR and exposed samples. # *p* < 0.05.

Immunolocalization of cardiac α-actinin and of Troponin T was conducted on whole-mount samples with the aim of maintaining unaltered the sarcomere structure in the spatial and multi-layered arrangement of differentiated cardiomyocytes within the 3D complex organization of the EBs. In CTR samples, immunofluorescence images displayed typical striated sarcomere-specific pattern (Fig. 2A, C).

In ATO-exposed EBs, cardiomyocytes with altered patterns co-exist with cardiomyocytes with regularly striated patterns (Fig. 2B, D). To address the contribution of the latter to the maintenance of the EB beating capacity, we employed a newly developed bioinformatic tool^30^ capable to extract from images of the sarcomere measurable values related to its organization. The SOTA algorithm^30^ was applied on samples immunostained with cardiac α-actinin, marker of the sarcomeric Z-lines.

The irregular and disorganized cardiac α-actinin fluorescent pattern observed at all doses was highlighted by significantly (*p* < 0.001) lower values of the SOTA’s Haralick, Gabor and Fourier parameters (Fig. 2E) specific for assessing the texture features, the edge and the repetitiveness of a structure, respectively.

Instead, the three scores were not significantly different (*p* > 0.05) in the comparison among organized sarcomeres of CTR and ATO-exposed samples (Fig. 2F). In cardiomyocytes exposed to all ATO doses, the parameter that describes the distance between two Z bands (length) was significantly lower (*p* < 0.05) in organized sarcomeres than the corresponding values obtained in CTR samples, indicating shorter sarcomeres (Fig. 2G). To the contrary, sarcomere width (Z band height) of organized sarcomeres in the comparison between CTR and ATO-exposed cardiomyocytes was not significant different (*p* > 0.05) (Fig. 2H).

### Expression profiles of sarcomeric proteins

**Figure 3:**
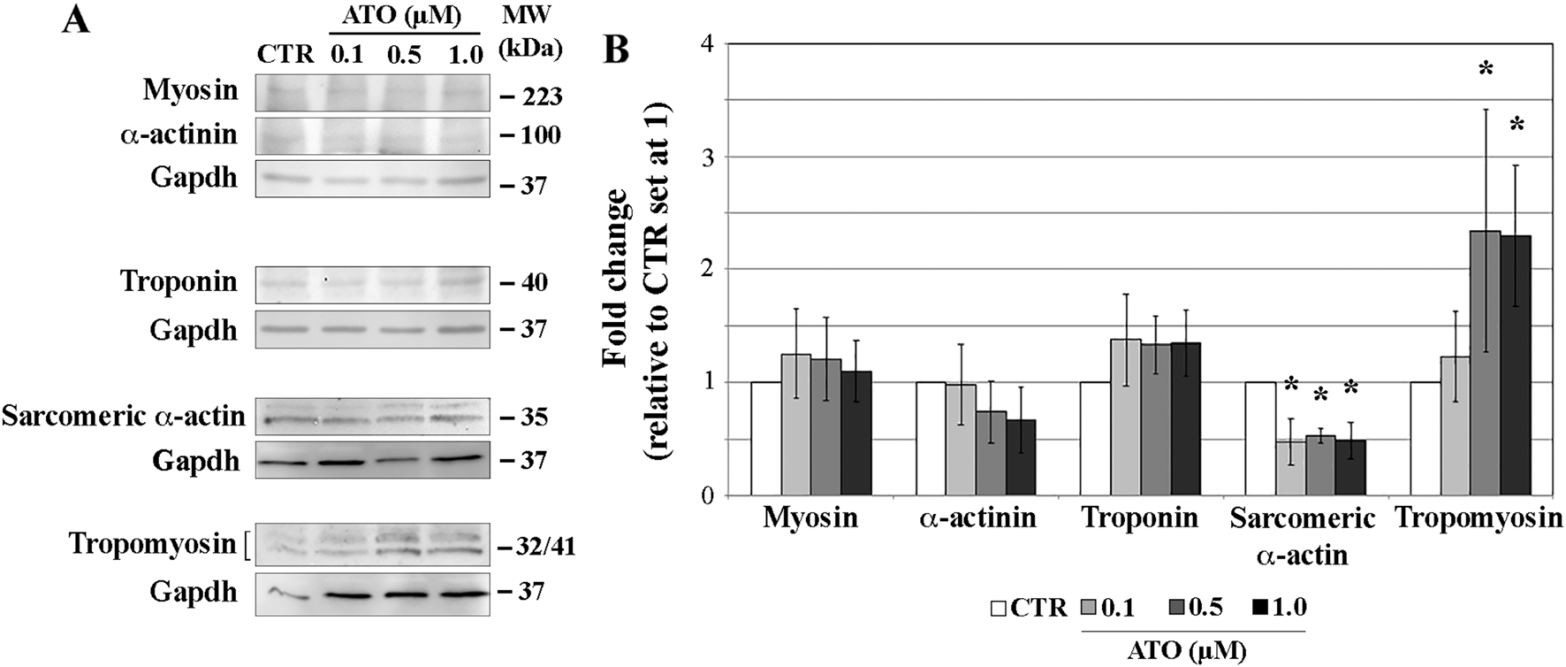
Western blotting analysis of myosin, cardiac α-actinin, troponin, tropomyosin and α-actin sarcomeric proteins (**A**) and the results of their quantitation (**B**). *p < 0.001.

Since disruption of sarcomere organization correlates with skewed quantitative ratio of sarcomeric proteins^14,31^, Myosin, cardiac α-actinin, Troponin T, Tropomyosin and sarcomeric α-actin expression profiles were quantitatively evaluated (Fig. 3A and B).

The quantity of Myosin, cardiac α-actinin and Troponin T was similar (*p* > 0.05) in CTR and exposed samples at all ATO doses. Instead, the expression of the sarcomeric α-actin was lower (0.47-, 0.53-, 0.49-fold in 0.1, 0.5 and 1.0 µM ATO-exposed EBs, respectively; *p* < 0.001) and that of Tropomyosin was higher (2.4- and 2.3-fold at 0.5 and 1.0 µM ATO exposure, respectively; *p* < 0.001) compared to CTR. This altered expression determines a skewed ratio of Tropomyosin at 0.5 and 1.0 µM ATO and of sarcomeric α-actin at all doses, whereas for the other three sarcomeric proteins the ratio was consistent with that of CTR samples and of foetal cardiomyocytes used as reference^14,32^ (Table 1).

**Table 1:** Sarcomeric protein ratio referred to Troponin (set at 1) of foetal, CTR and ATO-exposed cardiomyocytes.

Sample	Myosin	α-actinin	Troponin	α-actin	Tropomyosin
Foetal cardiomyocytes	1.5	2.9	1.0	7.0	1.0
CTR	1.5	2.9	1.0	7.0	1.0
0.1 µM	1.5	2.9	1.0	3.3	1.0
0.5 µM	1.5	2.9	1.0	3.6	1.0
1.0 µM	1.5	2.9	1.0	3.3	2.3

The observed alteration of sarcomere protein components suggests a possible association with change in cardiomyocyte morphology, specifically shape and area.

### Quantitative metrics of cardiomyocyte shape and area

**Figure 4:**
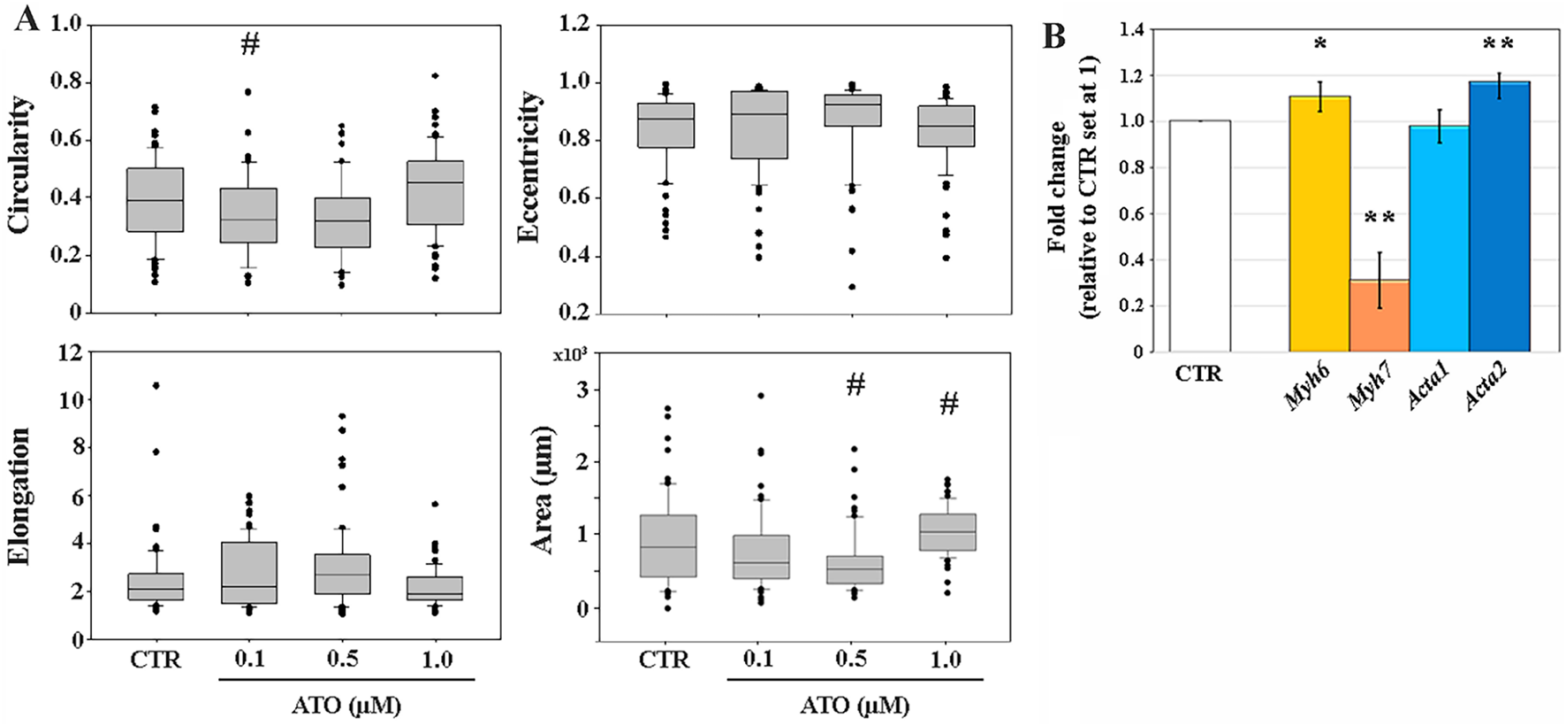
(**A**) Circularity, eccentricity, elongation and area values of control (CTR) and ATO-treated cardiomyocytes. The horizontal line within the box is the median, whereas the upper and lower lines are the 25th percentile and 75th percentile, respectively. The black dots are out-layer values. # *p* < 0.05. (**B**) Fold change of the expression profile of *myh6*, *myh7*, *acta1* and *acta2* genes, calculated for 1.0 µM ATO-samples. *p < 0.05; **p < 0.001.

Morphological features of single cardiac cells, immunostained with cardiac α-actinin, were determined in both CTR and ATO-exposed sample, obtained after EBs disaggregation. Based on single cell perimeter (Fig. 2S), circularity, eccentricity, elongation and area were calculated applying the SOTA algorithm. Eccentricity and elongation were not different between CTR and exposed samples at all ATO doses, whereas circularity was lower (0.87-fold change, *p* < 0.05) at 0.1 µM. Cardiomyocyte area is 0.64-fold lower at 0.5 µM (*p* < 0.05), while it is 1.24-fold higher (*p* < 0.05) at 1.0 µM ATO compared to that of CTR (Fig. 4A).

Since changes in cardiomyocytes shape and area have been associated to variations in the expression of genes coding for actin and myosin isoform filaments^33^, transcript levels *acta1* and *acta2* (coding for the alpha-1-skeletal muscle actin and alpha-2-smooth muscle actin, respectively), and of *myh6* and *myh7* (coding for the α- and β-heavy myosin chains, respectively) were analyzed in 1.0 µM ATO samples (Fig. 4B). Transcript expression of *acta1* was similar (*p* > 0.05), whereas that of *acta2* was 1.18-fold higher (*p* < 0.001) in treated compared to CTR cardiomyocytes. The number of gene transcripts of *myh6* significantly increased (1.11-fold) (*p* < 0.05), whereas that of *myh7* significantly decreased (0.31-fold change) (*p* < 0.001) in ATO-exposed samples, leading to a *myh6*/*myh7* fourfold altered ratio.

The modification of the cell shape and area after ATO exposure suggested the investigation of possible changes in the volume occupied by cardiomyocytes in the 3D structure of the EBs.

### Quantitative metrics of cardiomyocyte volume in the 3D context of the EBs

**Figure 5:**
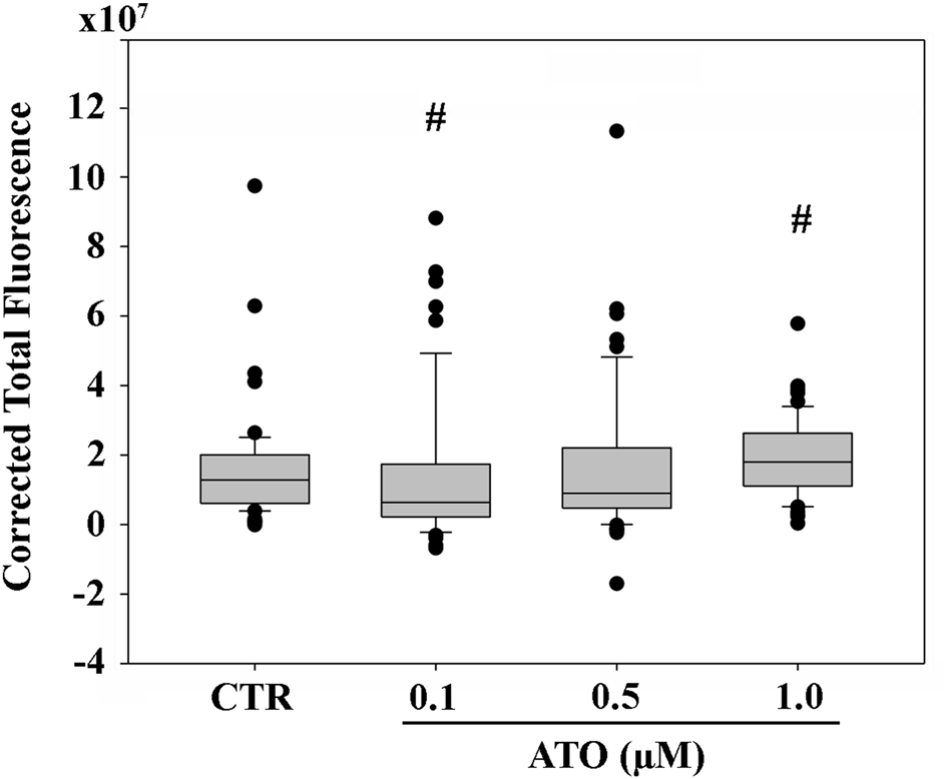
Corrected Total Fluorescence of cardiac α-actinin signals corresponding to the volume occupied by the cardiomyocyte tissue in EB core samples. The horizontal line in the box is the median, whereas the upper and lower lines are the 25th percentile and 75th percentile, respectively. The black dots are out-layer values. CTR, control; # *p* < 0.05.

We used a stereology approach, as it is applied in 2D histological sections, to quantify the volume occupied by the cardiomyocyte tissue in the EBs 3D structure. To this end, the cardiac α-actinin Corrected Total Fluorescence (CTF) was evaluated on 2D images generated through the overlap of all the focal planar planes present in a core sample (Video 5S and Fig. 3S). The results obtained are summarized in Fig. 5. The CTF value of 0.1 µM ATO-exposed samples was significantly lower (0.3-fold-change; *p* < 0.05) compared to that of CTR. On the contrary, in 1.0 µM ATO-exposed, the CTF value was significantly 1.3-fold higher (*p* < 0.05). The CTF value of samples exposed to 0.5 µM ATO was similar compared to that of CTR (*p* = 0.1352).

### Expression profiles of gap junction genes and proteins

**Figure 6:**
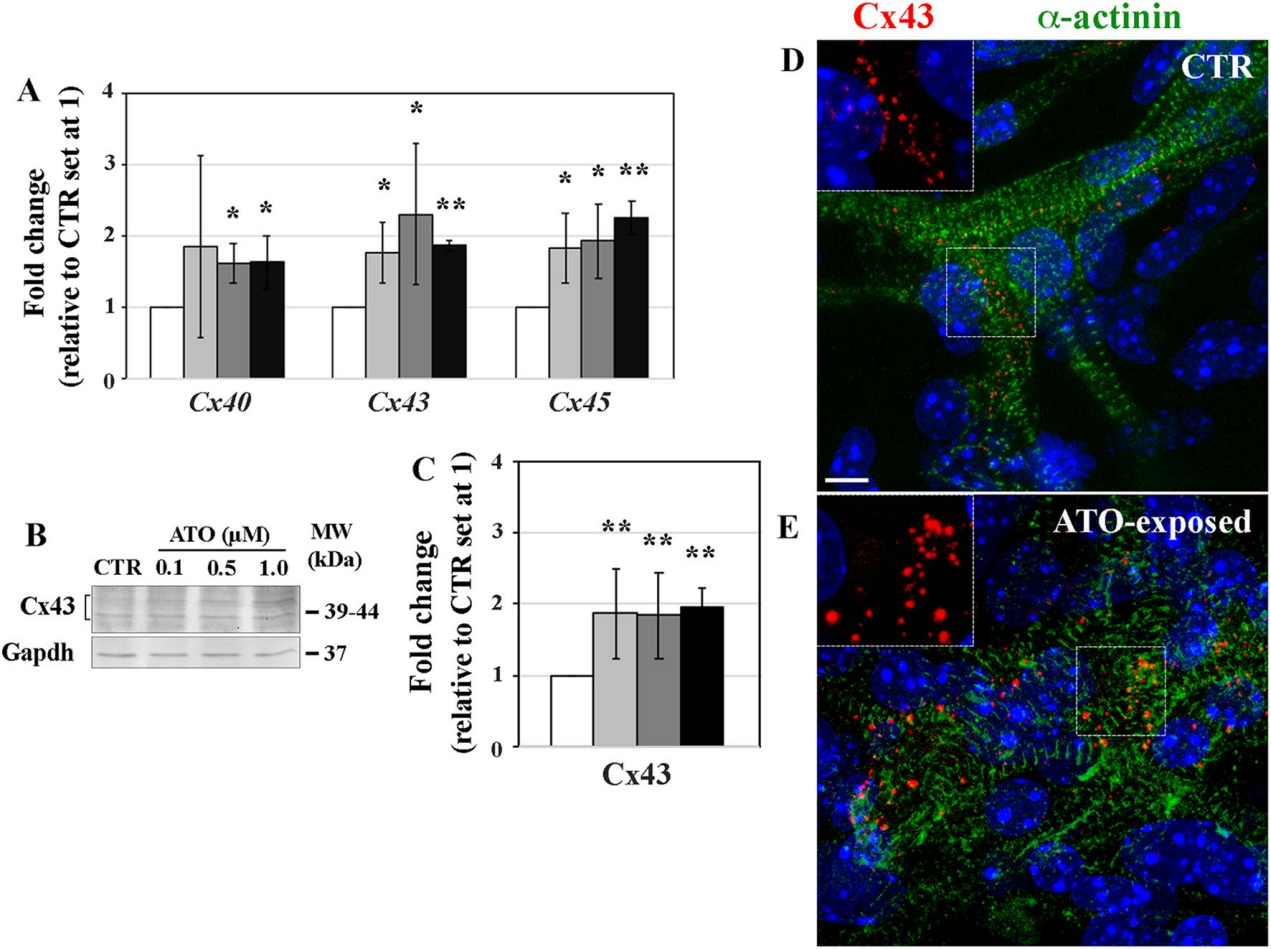
Expression profile of genes coding for the major connexin isotypes of control (CTR) and ATO-treated cardiomyocytes (**A**). Western blotting analysis of Connexin 43 (Cx43) protein (**B**) and its quantitation (**C**). *p < 0.05; **p < 0.001. Whole mount immunofluorescence localization of Cx43 (magnification in the inset) and of α-actinin proteins in CTR (**D**) and in 1.0 µM ATO-exposed (**E**) cardiomyocytes. Nuclei are stained with DAPI (blue). Bar: 5 μm.

At 0.1 µM ATO, *Cx40* gene transcript quantification did not show significant (*p* > 0.05) differences, whereas the expression of *Cx43* and *Cx45* was significantly higher compared to CTR (1.7- and 1.8-fold, respectively; *p* < 0.05) (Fig. 6A). In cardiomyocytes exposed to 0.5 μM ATO, *Cx40*, *Cx43* and *Cx45* gene transcripts were 1.6-, 2.3- and 1.9-fold up-regulated respectively (*p* < 0.05), compared to CTR cardiac cells. Similarly, in cardiomyocytes treated with the highest ATO dose, *Cx40, Cx43* and *Cx45* transcripts showed 1.6, 1.8- and 2.2-fold change increase (*p* < 0.001; *p* < 0.05), respectively (Fig. 6A).

The expression of Cx43, the most abundant connexin of the heart, was also determined by Western blotting and mapped in situ by immunofluorescence on whole-mount EBs. When compared to CTR (Figs. 6B, C, D and E) cardiomyocytes exposed to all ATO doses displayed substantial increase of Cx43 protein expression (1.8-, 1.7- and 1.9-fold; *p* < 0.001) (Figs. 6B and C) with large and irregularly distributed foci on cardiac α-actinin-positive cardiomyocytes (Fig. 6E).

To understand whether ATO exerts general toxicity on the cytoskeleton or gap junction protein components, Cx43 and phalloidin (marker of cytoskeletal actin) fluorescent signals were quantified in epithelial-like cells, localized at the borders of the 3D cell mass structure. As shown in Fig. 4S, no significant differences (*p* > 0.05) were detected between CTR and 1.0 µM ATO-exposed cells, the highest concentration used in this study, suggesting that the observed effects of ATO may be considered cardiomyocyte-specific.

## Discussion

Making use of a mESC differentiation platform, the aim of this study was to investigate the detrimental effects of ATO on the perinatal-like cardiomyocyte component of the EBs, with a focus on cell organization and function. To the best of our knowledge, this is the first report that combines a bioinformatic analysis with cellular and molecular tools to obtain functional and structural quantitative metrics of the impairment within the 3D complex cell organization of the EBs. Our results evidence that cardiomyocytes exposed to 0.1, 0.5 or 1.0 μM ATO for 72 h display impaired sarcomere structure, gap junction protein expression, cell size and shape and beating activity, detrimental effects that can be considered cardiomyocyte-specific. In fact, quantification of phalloidin and of Cx43 fluorescent signals on epithelial-like cells present at the borders of the EBs showed no significant differences between CTR and 1.0 µM ATO-exposed samples, an indication that a general toxicity on cytoskeleton or gap junction components is unlikely. Instead, ATO may exert cell-specific effects, like those observed in cardiomyocytes. Exposure to the toxicant affects the kinematic and dynamic cardiomyocyte parameters examined at all concentrations tested, highlighting contractile dysfunction. In particular, at the highest 1.0 µM concentration, increased chronotropy is accompanied by decreased inotropy and ergotropy, suggesting an unfavorable ‘force-frequency relationship’^29^ in comparison to CTR. Interestingly, altered beating activity is not paralleled by variations in the ATP content, suggesting that the regulation of ATP homeostasis may not be affected by the presence of ATO.

Impaired cell-to-cell coupling, associated with altered gap junctions, may contribute to contractile dysfunction. To this regard, significant enhancement of the expression profiles of *Cx40*, *Cx43* and *Cx45* connexin genes and of Cx43 protein, paralleled by large Cx43 immunofluorescence signals, might contribute to the remodeling of gap junctions leading to abnormal cardiomyocyte conduction properties. In support of our hypothesis, structural model studies showed that arsenic can directly bind to Cx43 channels potentially causing conformational changes in their structure^34^. However, on the basis of our results, we cannot determine whether enhancement of connexins expression is a result or a cause of changed contraction, or even an unrelated effect.

The alteration of the contractile activity may also be correlated to the remodeling of sarcomere organization, the cell’s force-generating unit. In fact, whole-mount immunostaining applied to the 3D structure of the EBs evidenced the existence of two different populations of cardiomyocytes: 1) those with regular striated sarcomere pattern and 2) those with disorganized and/or disoriented sarcomeres, with partial or complete loss of the regular striated pattern. Cardiomyocytes with altered sarcomere are present within the whole volume of ATO-exposed EBs (from the top to the bottom of the cell mass; data not shown), as well as cardiomyocytes with regular striated sarcomere pattern. Thus, we reasonably exclude that the coexistence of these two populations of cardiomyocytes is related to a penetration limit of ATO. The presence of two distinct populations of cardiomyocytes might be related to the heterogeneity of the differentiation status of cardiomyocytes within the EBs cell population^17^.

The application of a bioinformatic approach^30^ allowed a quantitative metrics of cardiomyocyte sarcomere texture within the 3D EB complex organization, revealing that organized sarcomeres of ATO-exposed cells had the same parameters as those of CTR cardiac cells, except for sarcomere length, which is significantly reduced. The shortening of the distance between the Z bands may correspond to a more contracted cardiomyocyte phenotype, a condition known to be associated to impaired contraction ability^35^. In cardiomyocytes with disorganized sarcomeres, all the texture quality parameters dramatically drop. The loss of a correct sarcomere structure is a typical feature of some heart failure pathologies (cardiac hypertrophy and cardiomyopathies)^36–38^. Moreover, sarcomere disruption is frequently correlated to changes of the expression of both contractile and regulatory proteins^38,39^. We observed that the expression of sarcomeric α-actin and Tropomyosin proteins is altered and we propose that the skewed protein ratio of two (in our experimental conditions) proteins of those making up the sarcomeres may contribute to disrupt their organization, as also reported previously^14,31,40^. Also, we cannot exclude that ATO might interfere with the translation of sarcomeric proteins, their turnover, or cause their post-translational modifications. To this regard, ATO, through the production of reactive oxygen and nitrogen species in exposed cells^41,42^, can indirectly provoke oxidation or nitrosylation of the sarcomeric proteins, causing their structural changes^43^. These alterations might severely impair the stability of the proteins present in the sarcomeric apparatus, damaging those interactions necessary to guarantee the maintenance of the sarcomere structure and thus their proper contraction ability.

Changes in both cardiac Troponin I expression and contractile parameters were also observed in human-induced pluripotent stem cells-derived cardiomyocytes (hiPS-CMs)^44^. In particular, the impairment of the kinematic properties was dependent on the concentration and on the time of exposure to the toxicant, with a gradual (after 5–6 days) or a rapid (within 4–12 h) decrease in the contraction velocity recorded at 3.0 μM or 10.0 µM ATO concentration, respectively^44^. A dose-dependent alteration of the kinematics and dynamics features was reported when ATO, at 0.1, 0.5 and 1.0 µM concentrations, was present throughout the 15 days of differentiation from mouse ESCs to cardiomyocytes^14^. The altered contractile parameters were paralleled by reduced expression of α-actinin, Myosin, Troponin T sarcomeric and Cx43 gap junction proteins^14^. Interestingly, the same concentrations of ATO elicited different cellular responses in perinatal-like cardiomyocytes when present throughout the 15 days of their differentiation from ESCs or for 3 days after their differentiation (this study). In summary, ATO prompts cellular reactions that might depend on the species, on the cell type from which cardiomyocytes are derived or even on the time when cells are exposed during differentiation.

Altered expression of *acta2* and *myh6*, *myh7* transcripts, whose impairment is associated to cardiomyocyte cell enlargement in the adult heart^37,45^, was found after 1.0 µM ATO exposure. In the mouse ventricle, β-MHC, coded by *myh7*, is the predominant isoform in the developing heart, then replaced by α-MHC, coded by *myh6*, after birth. The *myh6/myh7* ratio is important during cardiac development for the acquisition of proper cardiomyocyte mechanical performance and contraction efficiency^46^ and its alteration is indicative of structural changes in the sarcomere structure and associated to fetal postnatal cardiac hypertrophy^45,46^. The altered expression of all these cytoskeleton components observed after ATO exposure might be involved in the impairment of the kinematic and dynamic properties, but also in reshaping cell morphology. As revealed by our bioinformatic analysis, both the cell area and shape and, in turn, the volume occupied by the cardiomyocyte tissue in the 3D structure of the EBs increase at the highest concentrations tested. Recently, Ceja-Galicia and collaborators showed that adipose cells exposed to arsenic permanently undergo cell size modification, induced by a reorganization of the cell cytoskeleton components^47^.

In conclusion, we exploited the characteristic features of ESCs to give rise to differentiated cardiomyocytes whose organization and function were studied within the 3D complex structure of the EBs. For the first time, coupling whole-mount immunofluorescence and molecular techniques with image analysis and bioinformatics tools we quantified the impairment of the sarcomere organization and of cardiomyocytes size and shape induced by ATO, correlating the remodeling of the cytoskeleton with the beating function. We believe that our methodological approach may represent an important contribution to the investigation of cardiac pathologies, by combining the differentiation of different types of pluripotent stem cells, e.g., induced pluripotent stem cells, into cardiomyocytes with bioinformatic image analysis performed on whole EBs.

## Methods

### Cell lines

R1 mESCs (kindly provided by Dr. Nagy, Samuel Lunenfeld Research Institute, Mount Sinai Hospital, Toronto, Ontario, Canada) and mouse STO-SNL2 cells (STO, American Type Culture Collection CRL-2225) were cultivated as previously described^48^.

### Differentiation of mESCs into cardiomyocytes

R1 mESCs were induced to differentiate into embryoid bodies (EBs) by removing the Leukemia Inhibitory Factor from the culture medium (differentiation medium), using the hanging drop method^24^. Briefly, for EBs formation, about seventy 20 µL droplets of culture medium containing 10^3^ mESCs were plated on the lid of p55 Petri dishes. On day 3 of culture, the developing EBs were transferred on 0.1% agarose-coated tissue dishes (Corning) and from day 5, about 5–8 EBs were plated in single 1.9 cm^2^ well or 2 EBs in single 0.3 cm^2^ well or onto 22 mm gelatin-coated 35 mm glass bottom dish (Greiner Bio-One) and cultivated for up to 15 days.

### ATO preparation and treatment

ATO (Sigma, cat. n. 11,099) was dissolved in 0.1 N NaOH in milliQ water (Millipore) to a final concentration of 100 µM. This solution was added to the culture medium on day 15 of differentiation to a final concentration of 0.1, 0.5 or 1.0 µM. As control samples, cells were cultured in the presence of 0.01 N NaOH (CTR). ATO or NaOH were left in the culture medium for 72 h, until day 18 of differentiation. Three independent experiments were performed for each of the assays described below.

### Contraction assays

About 20 EBs were plated onto 22 mm gelatin-coated Glass Bottom Dish (WillCo Wells), cultured up to day 15 and then exposed for 72 h to 0.1, 0.5 or 1.0 µM ATO. Then, dishes were transferred into the culture chamber of a Nikon BioStation IM, at 37 °C and 5% CO_2_, for video recording. For each experiment, AVI videos of the beating syncytia were recorded from 10 randomly chosen CTR or ATO-exposed samples, using the Snagit software and further analyzed with the Video Spot Tracker (VST) program used to track the motion of spots in AVI videos. Videos were then processed as previously described^27,28^. Briefly, 12 markers were positioned on randomly-chosen beating cardiomyocytes onto the first video frame. Then, for each marker, the spatial coordinates x and y, expressed in [pixel], and the temporal coordinate t, expressed in [s], were registered frame by frame. Using an image processing algorithm developed by one of us (L.F., see Fassina et al.^29^), the marker trajectories (characterized by vectors describing the movement, e.g., displacements and velocities) were mathematically calculated. Chronotropy was measured by counting the displacement peaks during the contraction movement; dynamic and kinematic inotropies were evaluated in terms of contraction force by the Hamiltonian mechanics (where force is demonstrated to be the gradient of total energy) and in terms of the maximum contraction velocities, respectively; ergotropy (consumption of energy to sustain the contraction movement) was estimated as mean kinetic energy of the beating syncytia.

### ATP quantitation assay

Cellular ATP content was determined using the ATPLite 1 step Luminescence ATP Detection Assay (PerkinElmer), following the manufacturer’s instructions. Briefly, 9 EBs for CTR or 9 for each ATO-exposed samples were washed twice with 100 µl of PBS, lysed with 50 µl of lysis buffer *per* well in a 96-well microplate (PerkinElmer), and shaked for 2 min at 700 rpm with an orbital shaker. Fifty µl of substrate solution was then added and incubated for 10 min in the dark. Luminescence was measured with a luminometer (Perkin Elmer Victor 2). Each sample was measured in triplicate. Three independent experiments were performed.

### Three-dimensional (3D) whole-mount immunofluorescence

Following 15 days of differentiation, about 12 EBs, plated onto 22 mm gelatin-coated Glass Bottom Dish (WillCo Wells), were exposed for 72 h to 0.1, 0.5 or 1.0 µM ATO. On day 18, CTR and ATO-exposed EBs were washed twice with 1X PBS and fixed in 4% cold paraformaldehyde in 1X PBS for 24 h. EBs were then permeabilized with 1% Triton X-100 in 1X PBS (PBT) for 30 min for 3 times and then blocked for 1 h with 10% FCS, 0.2% sodium azide in 1X PBS (Blocking buffer) at room temperature. EBs were washed twice in blocking buffer and incubated with mouse anti-cardiac α-actinin (1:800; Sigma) or with anti-mouse cardiac isoform of Troponin T (1:200; Thermo Fisher Scientific), and anti-rabbit Connexin 43 (Cx43; 1:75; Cell Signalling) primary antibodies, diluted in 1% FCS, 0.4% Triton X-100 (PBS-MT), 0.2% sodium azide in 1X PBS, for 24 h, at 4 °C on gentle rotation. In parallel, EBs were stained with TRITC-conjugated phalloidin (1:1000; Sigma). Then, EBs were rinsed 3 times for 45 min in 1% Triton X-100, 10% FCS in 1X PBS, 3 times for 10 min in 1X PBT, 3 times for 45 min in 1% FCS, 0.2% sodium azide in 1X PBS and 3 times for 10 min in 1X PBT. EBs were then incubated for 40 h at 4 °C on gentle rotation device with secondary antibodies diluted in Blocking buffer [Alexa fluor 488-conjugated anti-mouse IgG 1:500 (Molecular Probes); Alexa fluor 647-conjugated anti-rabbit IgG (Molecular Probes)]. After 3 washes with PBT, nuclei were counterstained with 0.2 µg/ml DAPI for 2 h and mounted in VECTASHIELD Mountain Medium (Vector Labs). All images were acquired with a 40X oil immersion objective with a Leica TCS SP8 confocal microscope.

Quantification of phalloidin and Cx43 immunostaining signals in non-cardiomyocyte cells was performed using Fiji software (http://imagej.nih.gov/ij/). Briefly, 50 regions of interest (ROIs) for CTR and 50 for 1.0 µM ATO-exposed samples of 2500 µm^2^ for phalloidin or of 0.968 µm^2^ for Cx43 were drawn and the fluorescent mean intensity value recorded.

### Protein extraction and western blotting analysis

On day 18, after 72 h ATO exposure, a total of 300 EBs for CTR or 300 for each ATO-exposed samples were washed two times with 1X PBS and collected by centrifugation at 500 rpm for 5 min. CTR and ATO-exposed cells were lysed in 50 mM Tris–HCl pH 8, 150 mM NaCl, 0.02% sodium azide, 1% Triton X-100 and 100 mg/ml PMSF. The concentration of the proteins was assayed using the Bradford method with a spectrophotometer (Bio-Rad). Samples were aliquoted and stored at −80 °C until usage.

Ten µg proteins were separated on 8–12% polyacrylamide gels and transferred on membranes (BioRad), overnight (40 V, 4 °C). Membranes were blocked and incubated with primary antibodies, as reported in Table 1S. After washes, the appropriated secondary antibodies were used to reveal the primary antibodies for 30 min at 37 °C, in agitation (Table 1S). Chemiluminescent detection was performed using the Westar ηC (Cyanagen), according to the manufacturers’ instructions. The blots were imaged with ChemiDoc XRS system (Bio-Rad) and acquired with Quantity One software (Bio-Rad). Densitometric intensities of the bands were determined with Image J software (National Institute of Health, http://imagej.nih.gov/ij/).

For the detection of different proteins on the same samples, the membranes were incubated with a stripping-buffer, containing 2% SDS, 62.5 mM Tris HCl pH 6.8 and 0.8% β-mercaptoethanol (Sigma), for 45 min at 50 °C in agitation. Then, the membranes were washed in tap water for 5 min in agitation and three more times using TBS-T for 10 min. At the end of the stripped procedure, membranes were reused for the detection, as previously described.

### RNA extraction, reverse transcription and quantitative Real-Time PCR

On day 18, after 72 h ATO exposure, RNA was extracted using the GenElute Mammalian Total RNA Kit (Sigma, according to the manufacturer’s instruction) from about 250 EBs for CTR or for each 0.1, 0.5, 1.0 µM ATO-exposed samples. All traces of DNA contamination were eliminated using the On-column DNaseI Digestion Kit (Sigma, according to the manufacturer’s instruction). Reverse transcription and quantitative Real-Time PCR reactions were performed as previously described^14^. The sequences of specific primers used are reported in Table 2S. β-2-microglobulin gene expression was used for sample normalization^14^.

### EBs dissociation and single-cell immunofluorescence

Cardiomyocytes for single-cell immunofluorescence analysis were isolated from CTR, 0.1, 0.5 or 1.0 µM ATO-exposed samples, as described in Neri et al.^31^. Briefly, a total of 160 EBs (40 for CTR and 40 for each ATO concentration) were mechanically detached from the wells, centrifuged at 500 rpm for 5 min and resuspended in 1 ml of “low Ca^2+^-medium” (120 mM NaCl, 5.4 mM KCl, 5 mM sodium pyruvate, 20 mM glucose, 20 mM taurine, 10 mM HEPES) for 15 min at room temperature. Then, cells were dissociated in low Ca^2+^-medium, supplemented with 1 mg/ml collagenase and 30 mM CaCl_2_, for 30 min at 37 °C. EBs dissociation was completed by vortexing the suspension for 1 min, at high speed. Cells were then centrifuged at 500 rpm for 5 min, resuspended in culture differentiation medium with 0.1, 0.5 or 1.0 µM ATO and seeded on 0.1% gelatin-coated coverslips in 24-well culture dishes. Following attachment, cardiomyocytes were fixed with 4% cold paraformaldehyde in 1X PBS for 20 min at room temperature and maintained in 1X PBS at 4 °C until usage.

For immunostaining, cells were permeabilized with 0.1% Triton X-100 in 1X PBS, then incubated with anti-cardiac α-actinin (1:800 in 1X PBS; Sigma) primary antibody for 1 h at 37 °C, rinsed thrice with 1X PBS, and then in an AlexaFlour 488-conjugated anti-mouse IgG (1:500 in 1X PBS; Molecular Probes) 1 h at 37 °C. After three washes with 1X PBS, nuclei were counterstained with 0.2 µg/ml DAPI and mounted in VECTASHIELD Mountain Medium (Vector Labs). Control experiments with the secondary antibody only were also carried out.

### Bioinformatic analysis

The SarcOmere Texture Analysis (SOTA) algorithm developed by Sutcliffe et al.^30^ was employed to quantify the texture organization of the sarcomeres and their length and width, and the area, eccentricity, circularity, and elongation of cardiomyocytes.

### Sarcomere organization

EBs, immunostained for cardiac α-actinin as described above, were used to determine the sarcomere organization. Within their 3D structure, sixty single stack images, derived from three independent experiments (twenty for each experiment), of CTR or of each ATO-exposed sample were acquired with a 63X oil immersion objective plus 1.5 digital zoom with a Leica TCS SP8 confocal microscope. Stacks were obtained with axial distances of 0.5 μm.

Specific ROIs were drawn around portions of cardiomyocyte sarcomeres (Fig. 5S). Using the MATLAB® software (The MathWorks, Inc.), sarcomere organization was evaluated via a Fourier score (based on Fourier transforms), a Gabor score (based on Gabor filters), and a Haralick texture feature (Haralick correlation being an indicator of organization). In detail, the Fourier transform converts an image to the frequency domain to assess the repeating structure of the sarcomere; the Gabor filter is able to detect image edges, in our study the sarcomere’s edges; the Haralick correlation is calculated from the gray level co-occurrence matrix of an image^30^. Sarcomere length and width were also calculated following Sutcliffe et al.^30^.

For the calculation of Fourier Score, Gabor Score, Haralick Correlation, sarcomere length and width parameters, a total of 290 ROIs for CTR or for each of the three ATO concentrations were analyzed.

### Cardiomyocyte shape and area

Cardiomyocytes were dissociated and immunostained for cardiac α-actinin as described above. Fifty cardiomyocyte images of CTR or of each ATO-exposed sample were acquired with 100X oil immersion objective with an Olympus BX60 fluorescence microscope, captured with a DP72 camera (Olympus) and processed using cellSens 1.4.1 software.

The morphological parameter analyzed with SOTA were the cardiomyocyte area, eccentricity, circularity, and elongation as described in Sutcliffe et al.^30^. Specifically, the perimeter of each cardiomyocyte was delimited to determine its area along with the minor and major axis lengths. These four parameters were used to evaluate the following cell shape parameters:

1. Eccentricity is comprised between 0 and 1, where a value of 0 represents a circumference, a value greater than zero but less than 1 represents an ellipse.
2. Circularity equal to 1 represents a circumference; a circularity < 1 represents an ellipse.
3. Elongation is a form factor; an elongation equal to 1 represents a circumference; an elongation > 1 represents an ellipse.

For area, circularity, eccentricity, and elongation parameters, a total of 65 cells for CTR or for each of the three ATO concentrations were analyzed.

### Cardiomyocyte volume

EBs were immunostained for α-actinin, as described above. Stacks were obtained from at least 20 core samples for each experiment, for a total of 60 core samples for CTR and each of the three ATO concentrations. The acquisition of each core sample starts from the first visible nucleus on the top of a selected region of the EB and continues until the surface of the dish, to which cells are attached, thus acquiring for the entire thickness of the EB (Fig. 5S). Images were acquired with a 40X oil immersion objective with a Leica TCS SP8 confocal microscope. Stacks were obtained with axial distances of 0.5 μm. To determine the volume occupied by cardiomyocytes in the core samples, a stereology approach based on the extrapolation of 3D information from the 2D planar sections of a tissue was applied. The ImageJ software tool called SUM (https://imagej.nih.gov/ij/), able to sum the cardiac α-actinin-related pixel intensities of all optical sections along the Z direction of a core sample, was applied. Then, the Integrated Density (IntDen) tool was applied to calculate the integral of the fluorescence intensity in an area where it is not homogeneously distributed. Then, using an area in which cardiomyocytes were absent, the mean background value was evaluated with the same approach. Finally, the CTF was determined applying the following formula:

$${\text{CTF}} = \left( {\text{IntDen of EB core sample SUM}} \right) - {\text{n*}}\left( {\text{mean of background IntDen}} \right)$$

where n is the number of images of each single core sample.

### Statistics

All data are presented as means ± standard deviation (SD), except for the syncytium contractile properties that are expressed as mean ± 95% confidence interval for the differences between means. Data were analyzed by the one-way ANOVA (significance level of 0.05) followed by the post hoc LSD test.

## Supplementary Information

Supplementary Figure 1S.

Supplementary Figure 2S.

Supplementary Figure 3S.

Supplementary Figure 4S.

Supplementary Figure 5S.

Supplementary Original and uncropped images of Western blotting membranes of Figure 3.

Supplementary Legends.

Supplementary Table 1S.

Supplementary Table 2S.

Supplementary Video 1.

Supplementary Video 2.

Supplementary Video 3.

Supplementary Video 4.

Supplementary Video 5.
