# Supplementary figures and images for: Functional and structural phenotyping of cardiomyocytes in the 3D organization of embryoid bodies exposed to arsenic trioxide
Source: Sci Rep. 2021 Nov 30;11:23116. doi: 10.1038/s41598-021-02590-8 (PMC8633008; doi:10.1038/s41598-021-02590-8)

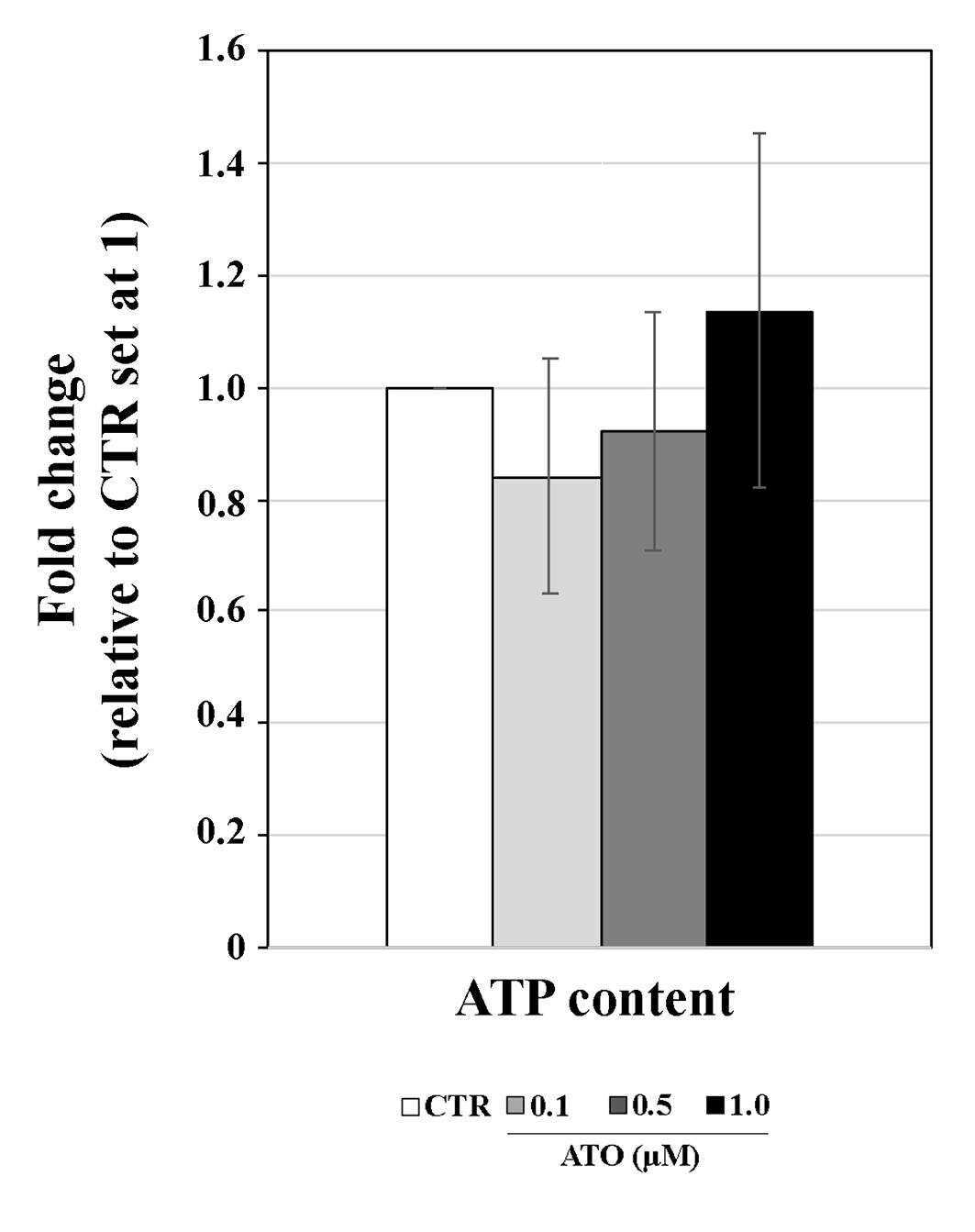

Supplement: Supplementary file 1 — Supplementary Figure 1S. [file 41598_2021_2590_MOESM1_ESM.tif]

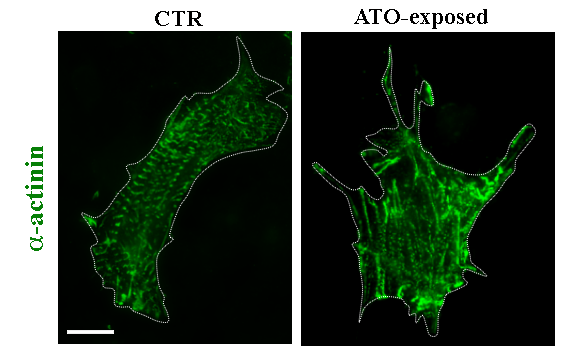

Supplement: Supplementary file 2 — Supplementary Figure 2S. [file 41598_2021_2590_MOESM2_ESM.tif]

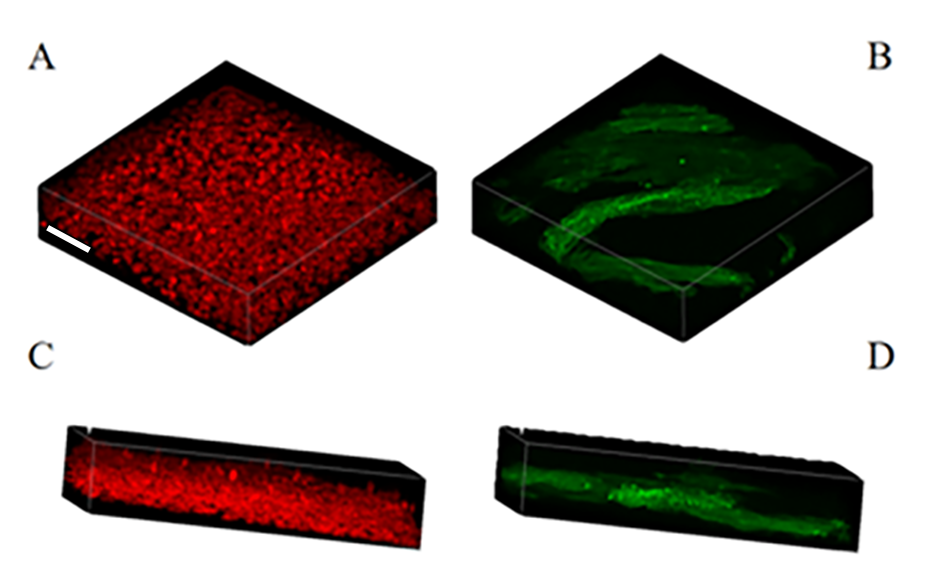

Supplement: Supplementary file 3 — Supplementary Figure 3S. [file 41598_2021_2590_MOESM3_ESM.tif]

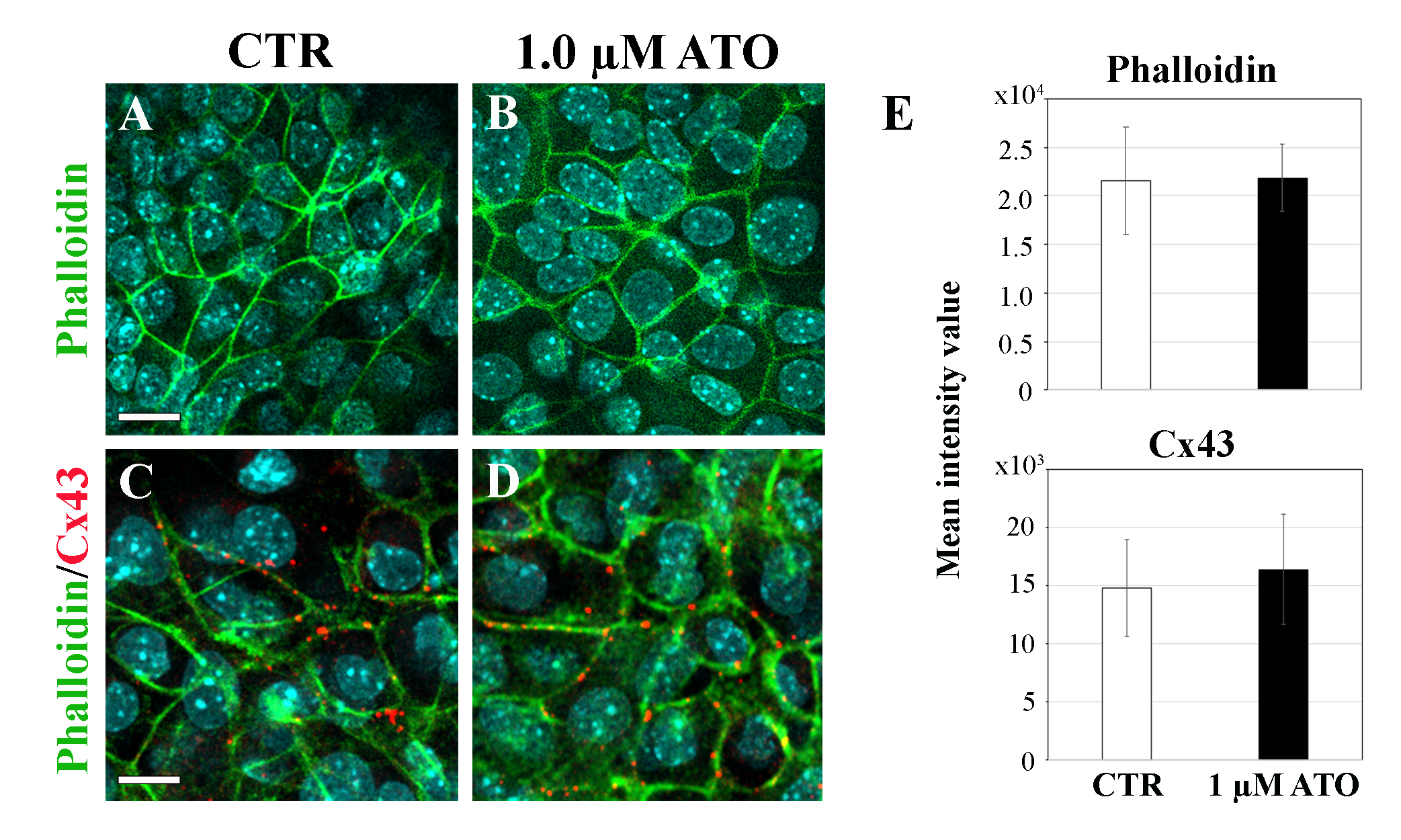

Supplement: Supplementary file 4 — Supplementary Figure 4S. [file 41598_2021_2590_MOESM4_ESM.tif]

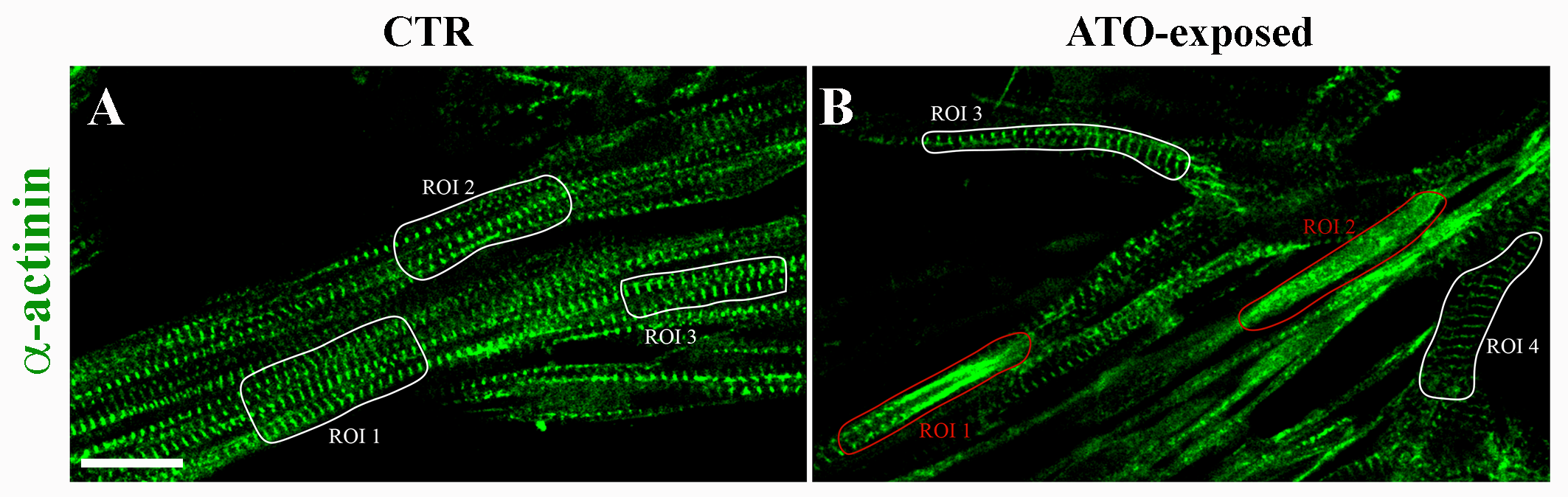

Supplement: Supplementary file 5 — Supplementary Figure 5S. [file 41598_2021_2590_MOESM5_ESM.tif]
