# Supplementary material for: Functional and structural phenotyping of cardiomyocytes in the 3D organization of embryoid bodies exposed to arsenic trioxide
Source: Sci Rep. 2021 Nov 30;11:23116. doi: 10.1038/s41598-021-02590-8 (PMC8633008; doi:10.1038/s41598-021-02590-8)
Supplement: Supplementary file 6 — Supplementary Original and uncropped images of Western blotting membranes of Figure 3. [file 41598_2021_2590_MOESM6_ESM.pdf]

Functional and structural phenotyping of cardiomyocytes in the 3D organization of embryoid bodies exposed to arsenic trioxide

Paola Rebuzzini[1,†,*], Cinzia Civello[1,†], Lorenzo Fassina[2,3], Maurizio Zuccotti[1,3,*] and Silvia Garagna[1,3,*]

[1] Laboratory of Developmental Biology, Department of Biology and Biotechnology "Lazzaro Spallanzani", University of Pavia, Via Ferrata 9, Pavia, Italy;

[2] Department of Electrical, Computer and Biomedical Engineering (DIII), University of Pavia, Via Ferrata 5, Pavia, Italy;

[3] Centre for Health Technologies (CHT), University of Pavia, Via Ferrata 5, Pavia, Italy.

[†] These authors contributed equally to the work

***Corresponding authors:**
Paola Rebuzzini
Laboratorio di Biologia dello Sviluppo
Dipartimento di Biologia e Biotecnologie 'Lazzaro Spallanzani'
Università degli Studi di Pavia
Via Ferrata 9, 27100 Pavia, Italy
Tel +39 0382 986323
Fax +39 0382 986270
e-mail: paola.rebuzzini@unipv.it

Maurizio Zuccotti
Laboratorio di Biologia dello Sviluppo
Dipartimento di Biologia e Biotecnologie 'Lazzaro Spallanzani'
Università degli Studi di Pavia
Via Ferrata 9, 27100 Pavia, Italy
Tel +39 0382 986323
Fax +39 0382 986270
e-mail: maurizio.zuccotti@unipv.it

Silvia Garagna
Laboratorio di Biologia dello Sviluppo
Dipartimento di Biologia e Biotecnologie 'Lazzaro Spallanzani'
Università degli Studi di Pavia
Via Ferrata 9, 27100 Pavia, Italy
Tel +39 0382 986323
Fax +39 0382 986270
e-mail: silvia.garagna@unipv.it

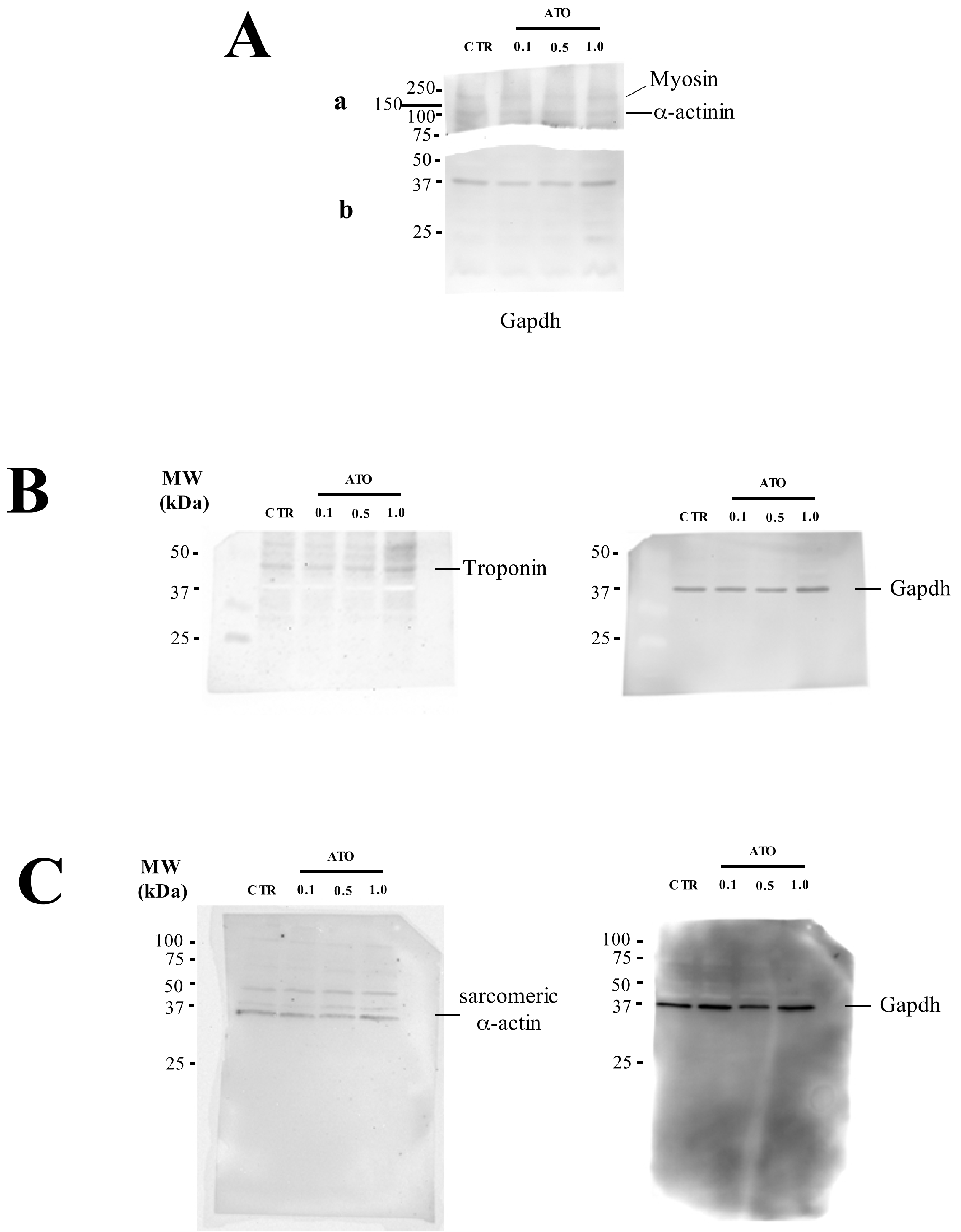
A
ATO
CTR 0.1 0.5 1.0
a
b
250
150
100
75
50
37
25
Myosin
α-actinin
Gapdh
B
MW (kDa)
ATO
CTR 0.1 0.5 1.0
50
37
25
Troponin
ATO
CTR 0.1 0.5 1.0
50
37
25
Gapdh
C
MW (kDa)
ATO
CTR 0.1 0.5 1.0
100
75
50
37
25
sarcomeric α-actin
ATO
CTR 0.1 0.5 1.0
100
75
50
37
25
Gapdh

D

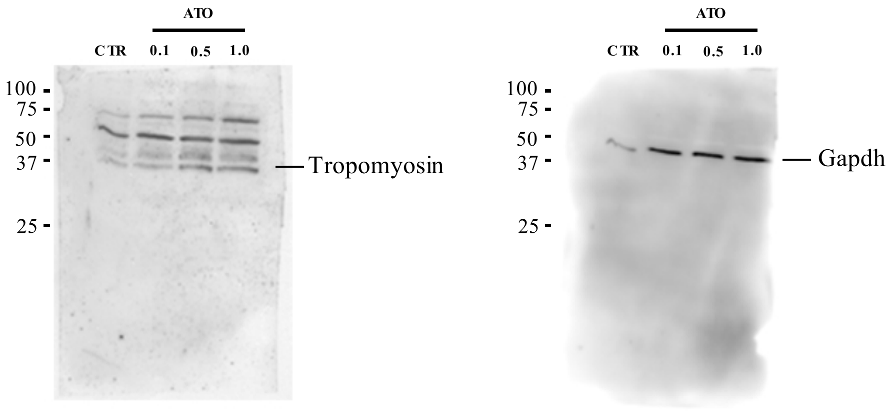

E

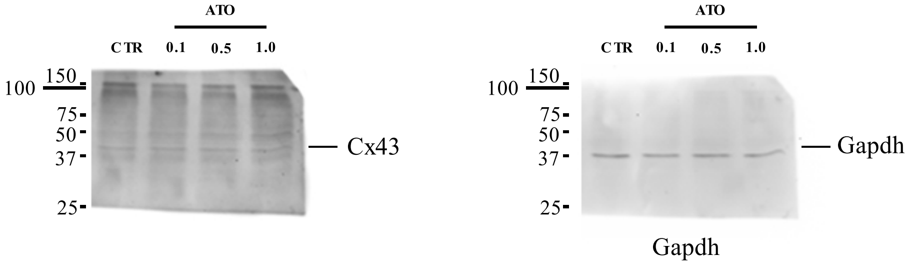

Original and uncropped images of Western blotting membranes of Figure 3.

After run and transfer, membrane **A** was cut in 2 parts, identified as "**a**" and "**b**".
Membrane "**a**" was hybridized with a mixture of anti-myosin and anti-α-ctininin antibodies, whereas "**b**" was hybridized with an anti-gapdh antibody.

Membranes **B**, **C**, **D** and **E** were firstly hybridized with anti-troponin (**B**), anti-sarcomeric α-actin (**C**), anti-tropomyosin (**D**) and anti-cx43 (**E**), respectively.
Then, membranes **B**, **C**, **D** and **E** were stripped and re-hybridized with an anti-gapdh antibody.
