# Supplementary material for: Functional and structural phenotyping of cardiomyocytes in the 3D organization of embryoid bodies exposed to arsenic trioxide
Source: Sci Rep. 2021 Nov 30;11:23116. doi: 10.1038/s41598-021-02590-8 (PMC8633008; doi:10.1038/s41598-021-02590-8)
Supplement: Supplementary file 7 — Supplementary Legends. [file 41598_2021_2590_MOESM7_ESM.pdf]

Functional and structural phenotyping of cardiomyocytes in the 3D organization of embryoid bodies exposed to arsenic trioxide

Paola Rebuzzini[1,†,*], Cinzia Civello[1,†], Lorenzo Fassina[2,3], Maurizio Zuccotti[1,3,*] and Silvia Garagna[1,3,*]

[1] Laboratory of Developmental Biology, Department of Biology and Biotechnology "Lazzaro Spallanzani", University of Pavia, Via Ferrata 9, Pavia, Italy;

[2] Department of Electrical, Computer and Biomedical Engineering (DIII), University of Pavia, Via Ferrata 5, Pavia, Italy;

[3] Centre for Health Technologies (CHT), University of Pavia, Via Ferrata 5, Pavia, Italy.

[†] These authors contributed equally to the work

***Corresponding authors:**
Paola Rebuzzini
Laboratorio di Biologia dello Sviluppo
Dipartimento di Biologia e Biotecnologie 'Lazzaro Spallanzani'
Università degli Studi di Pavia
Via Ferrata 9, 27100 Pavia, Italy
Tel +39 0382 986323
Fax +39 0382 986270
e-mail: paola.rebuzzini@unipv.it

Maurizio Zuccotti
Laboratorio di Biologia dello Sviluppo
Dipartimento di Biologia e Biotecnologie 'Lazzaro Spallanzani'
Università degli Studi di Pavia
Via Ferrata 9, 27100 Pavia, Italy
Tel +39 0382 986323
Fax +39 0382 986270
e-mail: maurizio.zuccotti@unipv.it

Silvia Garagna
Laboratorio di Biologia dello Sviluppo
Dipartimento di Biologia e Biotecnologie 'Lazzaro Spallanzani'
Università degli Studi di Pavia
Via Ferrata 9, 27100 Pavia, Italy
Tel +39 0382 986323
Fax +39 0382 986270
e-mail: silvia.garagna@unipv.it

Legends to supplementary figures

Figure 1S. ATP content, expressed as fold-change relative to control (CTR), of ATO-exposed EBs. Differences between CTR and exposed samples are not significative ($p>0.05$).

Figure 2S. Cardiac α-actinin positive cardiomyocytes isolated from control (CTR) and 1.0 μM ATO-exposed samples. Single cells are delimited for the evaluation of the shape and area following Sutcliffe et al.[30]. Bar: 5 μm.

Figure 3S. 3D representation of an embryoid body core sample. A) frontal and C) lateral view of nuclei (red) regardless of the cell type. B) frontal and D) lateral view of cardiomyocytes immunostained with cardiac α-actinin antibodies. Bar: 100 μm.

Figure 4S. Whole mount immunofluorescence localization of Phalloidin (A, B) and of Cx43 protein (C, D) in epithelial-like cells in CTR and 1.0 μM ATO-exposed EBs and (E) quantification of their fluorescent signals ($p>0.05$). Bar: 10 μm.

Figure 5S. Whole mount immunofluorescence localization of cardiac α-actinin. Examples of Regions of interest (ROI) delimiting regular striated patterns in A) control (CTR) (ROIs 1-3) and in B) 1 μM ATO-exposed (ROIs 3-4) samples. In B), ROIs 1 and 2 show altered cardiac α-actinin pattern. Bar: 5 μm.

Legends to Videos

Video 1S. Video of beating cardiomyocytes, recorded from a CTR sample.

Video 2S. Video of beating cardiomyocytes, recorded from a sample after 72h exposure to 0.1 μM ATO.

Video 3S. Video of beating cardiomyocytes, recorded from a sample after 72h exposure to 0.5 μM ATO.

Video 4S. Video of beating cardiomyocytes, recorded from a sample after 72h exposure to 1.0 μM ATO.

Video 5S. Overlap of all the focal planar planes present in a CTR core sample.
