# Supplementary material for: Functional and structural phenotyping of cardiomyocytes in the 3D organization of embryoid bodies exposed to arsenic trioxide
Source: Sci Rep. 2021 Nov 30;11:23116. doi: 10.1038/s41598-021-02590-8 (PMC8633008; doi:10.1038/s41598-021-02590-8)
Supplement: Supplementary file 8 — Supplementary Table 1S. [file 41598_2021_2590_MOESM8_ESM.pdf]

Functional and structural phenotyping of cardiomyocytes in the 3D organization of embryoid bodies exposed to arsenic trioxide

Paola Rebuzzini[1,†,*], Cinzia Civello[1,†], Lorenzo Fassina[2,3], Maurizio Zuccotti[1,3,*] and Silvia Garagna[1,3,*]

[1] Laboratory of Developmental Biology, Department of Biology and Biotechnology "Lazzaro Spallanzani", University of Pavia, Via Ferrata 9, Pavia, Italy;

[2] Department of Electrical, Computer and Biomedical Engineering (DIII), University of Pavia, Via Ferrata 5, Pavia, Italy;

[3] Centre for Health Technologies (CHT), University of Pavia, Via Ferrata 5, Pavia, Italy.

[†] These authors contributed equally to the work

***Corresponding authors:**
Paola Rebuzzini
Laboratorio di Biologia dello Sviluppo
Dipartimento di Biologia e Biotecnologie 'Lazzaro Spallanzani'
Università degli Studi di Pavia
Via Ferrata 9, 27100 Pavia, Italy
Tel +39 0382 986323
Fax +39 0382 986270
e-mail: paola.rebuzzini@unipv.it

Maurizio Zuccotti
Laboratorio di Biologia dello Sviluppo
Dipartimento di Biologia e Biotecnologie 'Lazzaro Spallanzani'
Università degli Studi di Pavia
Via Ferrata 9, 27100 Pavia, Italy
Tel +39 0382 986323
Fax +39 0382 986270
e-mail: maurizio.zuccotti@unipv.it

Silvia Garagna
Laboratorio di Biologia dello Sviluppo
Dipartimento di Biologia e Biotecnologie 'Lazzaro Spallanzani'
Università degli Studi di Pavia
Via Ferrata 9, 27100 Pavia, Italy
Tel +39 0382 986323
Fax +39 0382 986270
e-mail: silvia.garagna@unipv.it

Table 1S: Primary antibodies and block solutions used for Western Blotting analysis.

**Primary Antibody**	**Block composition/ Incubation time**	**Antibody dilution/ Incubation time**	**Source and catalogue number**
Mouse anti-heavy chain cardiac myosin	3% BSA in TBS-T*/ 30 min room temperature	1:1000 in TBS-T/ 2h at 37°C	Abcam Ab15
Mouse anti-sarcomeric α-actinin	3% BSA in TBS-T/ 30 min room temperature	1:1500 in TBS-T/ 2h at 37°C	SIGMA-ALDRICH A7811
Mouse Anti-sarcomeric actin (Alpha Sr-1)	5% BSA in TBS-T/ 1h room temperature	1:200 in 5%BSA in TBS-T Overnight 4°C	Santa Cruz Biotechnology Sc-58671
Mouse Anti-Troponin T cardiac isoform Ab-1, clone 13-11	3% BSA in TBS-T/ 30 min room temperature	1:1000 in TBS-T/ 2h at 37°C	Thermo Scientific MS-295-P1
Mouse Anti-tropomyosin (F-6)	5% BSA in TBS-T/ Overnight 4°C	1:250 in 5% BSA in TBS-T/ Overnight 4°C	Santa Cruz Biothecnology Sc-74480
Rabbit anti-Connexin 43	5% BSA in TBS-T/ 0.1%Tween-20 1h room temperature	1:1000 in 5% BSA, 0.1% Tween-20 in TBS-T/ Overnight 4°C	Cell Signalling 3512
Rabbit Anti-GAPDH	2% milk in PBS1X/ 30 min room temperature	1:2000 in 1% milk in TBS-T/ 2h at 37°C	GeneTex GTX100118

**Secondary Antibody**	**Antibody dilution/ Incubation time**	**Source and catalogue number**
Goat anti-mouse HRP-conjugated	1:20000 in 1% milk in PBS 1X/1h at 37°C	Molecular Probes F21453
Goat anti-rabbit HRP-conjugated	1:25000 in 2%BSA in TBS-T/1h at 37°C	Sigma A9169

*TBS-T: 20 mM Tris pH 7.5, 150 mM NaCl, 0.1% Tween 20
