# Supplementary material for: Functional and structural phenotyping of cardiomyocytes in the 3D organization of embryoid bodies exposed to arsenic trioxide
Source: Sci Rep. 2021 Nov 30;11:23116. doi: 10.1038/s41598-021-02590-8 (PMC8633008; doi:10.1038/s41598-021-02590-8)
Supplement: Supplementary file 9 — Supplementary Table 2S. [file 41598_2021_2590_MOESM9_ESM.pdf]

Functional and structural phenotyping of cardiomyocytes in the 3D organization of embryoid bodies exposed to arsenic trioxide

Paola Rebuzzini[1,†,*], Cinzia Civello[1,†], Lorenzo Fassina[2,3], Maurizio Zuccotti[1,3,*] and Silvia Garagna[1,3,*]

[1] Laboratory of Developmental Biology, Department of Biology and Biotechnology "Lazzaro Spallanzani", University of Pavia, Via Ferrata 9, Pavia, Italy;

[2] Department of Electrical, Computer and Biomedical Engineering (DIII), University of Pavia, Via Ferrata 5, Pavia, Italy;

[3] Centre for Health Technologies (CHT), University of Pavia, Via Ferrata 5, Pavia, Italy.

[†] These authors contributed equally to the work

***Corresponding authors:**
Paola Rebuzzini
Laboratorio di Biologia dello Sviluppo
Dipartimento di Biologia e Biotecnologie 'Lazzaro Spallanzani'
Università degli Studi di Pavia
Via Ferrata 9, 27100 Pavia, Italy
Tel +39 0382 986323
Fax +39 0382 986270
e-mail: paola.rebuzzini@unipv.it

Maurizio Zuccotti
Laboratorio di Biologia dello Sviluppo
Dipartimento di Biologia e Biotecnologie 'Lazzaro Spallanzani'
Università degli Studi di Pavia
Via Ferrata 9, 27100 Pavia, Italy
Tel +39 0382 986323
Fax +39 0382 986270
e-mail: maurizio.zuccotti@unipv.it

Silvia Garagna
Laboratorio di Biologia dello Sviluppo
Dipartimento di Biologia e Biotecnologie 'Lazzaro Spallanzani'
Università degli Studi di Pavia
Via Ferrata 9, 27100 Pavia, Italy
Tel +39 0382 986323
Fax +39 0382 986270
e-mail: silvia.garagna@unipv.it

Table 2S: Reverse and forward primer sequences used for qRT-PCR.

Gene	Forward Primers	Reverse Primers
Cx40	5’ CATACCTCGGAGTGCTGGTG 3’	5’ GGCCAAGGACCAAGGATACC 3’
Cx43	5’ CTCCTCCTGGGTACAAGCTG 3’	5’ AATTCGCCCAGTTTTGCTCG 3’
Cx45	5’ TCATCCTGGTTGCAACTCCC 3’	5’ CTGCCTTCTTGTCTGCCTCA 3’
Myh6	5’ ATAAAGGGGCTGGAGCACTG 3’	5’ AGGCAGGAAGAGGAGTAGCA 3’
Myh7	5’ CCAAGGGCCTGAATGAGGAG 3’	5’ GCAAAGGCTCCAGGTCTGAG 3’
Acta1	5’ AAGTCCTGCAAGTGAACAAGC 3’	5’ GTTGTCACACACAAGAGCGG 3’
Acta2	5’ ACTCTCTTCCAGCCATCTTTCA 3’	5’ AGCATAGAGATCCTTCCTGATGTC 3’
β2m	5’ GAATTCACCCCCACTGAGACT 3’	5’ TGCTTGATCACATGTCTCGAT 3’
